# Growth hormone treatment associates with improved circulating anti-aging protein Klotho and reduced arterial stiffness in children with CKD

**DOI:** 10.1093/ckj/sfaf231

**Published:** 2025-07-23

**Authors:** Stella Stabouli, Maren Leifheit-Nestler, Michael Föller, Martina Feger, Aysun K Bayazit, Anke Doyon, Lukasz Obrycki, Bruno Ranchin, Jun Oh, Dusan Paripovic, Germana Longo, Jerome Harambat, Otto Mehls, Anette Melk, Uwe Querfeld, Franz Schaefer, Dieter Haffner, the 4C Study Consortium and the ESPN CKD-MBD Working Group, Gerard Cortina, Klaus Arbeiter, Jiri Dusek, Jerome Harambat, Bruno Ranchin, Michel Fischbach, Ariane Zaloszyc, Uwe Querfeld, Jutta Gellermann, Sandra Habbig, Max Liebau, Matthias Galiano, Rainer Büscher, Charlotte Gimpel, Matthias Kemper, Jun Oh, Anette Melk, Daniela Thurn-Valassina, Anke Doyon, Elke Wühl, Franz Schaefer, Ulrike John, Simone Wygoda, Nicola Jeck, Birgitta Kranz, Marianne Wigger, Francesca Mencarelli, Francesca Lugani, Sara Testa, Giovanni Montini, William Morello, Enrico Vidal, Elisa Benetti, Luisa Murer, Ciara Matteucci, Stefano Picca, Augustina Jankauskiene, Karolis Azukaitis, Aleksandra Zurowska, Ilona Zagozozon, Dorota Drodz, Tomasz Urasinski, Mieczyslaw Litwin, Anna Niemirska, Lukasz Obrycki, Maria Szczepanska, Ana Texeira, Amira Peco-Antic, Dusan Paripovic, Giacomo Simonetti, Guido Laube, Ali Anarat, Aysun K Bayazit, Fatos Yalcinkaya, Esra Baskin, Nilgun Cakar, Oguz Soylemezoglu, Ali Duzova, Yelda Bilginer, Hakan Erdogan, Osman Donmez, Ayse Balat, Aysel Kiyak, Salim Caliskan, Nur Canpolat, Mahmut Civilibal, Cengiz Candan, Sevinc Emre, Alev Yilmaz, Harika Alpay, Gul Ozcelik, Sevgi Mir, Betul Sözeri, Ipek K Bulut, Nejat Aksu, Onder Yavascan, Yilmaz Tabel, Pelin Ertan, Ebru Yilmaz, Rukshana Shroff

**Affiliations:** 1st Department of Pediatrics, Aristotle University Thessaloniki, Hippokratio Hospital, Thessaloniki, Greece; Department of Pediatric Kidney Liver, Metabolic and Neurological Diseases, Hannover Medical School, Hannover, Germany; Department of Physiology, University of Hohenheim, Stuttgart, Germany; Department of Physiology, University of Hohenheim, Stuttgart, Germany; Department of Pediatric Nephrology, Cukurova University, Adana, Turkey; Division of Pediatric Nephrology, Center for Pediatrics and Adolescent Medicine, Heidelberg University Hospital, Heidelberg, Germany; Department of Nephrology, Kidney Transplantation and Hypertension, The Children's Memorial Health Institute, Warsaw, Poland; Pediatric Nephrology Unit, Hôpital Femme Mère Enfant, Hospices Civils de Lyon, Université de Lyon, Lyon, France; Department of Pediatric Nephrology, University Hamburg-Eppendorf, Hamburg, Germany; Nephrology Department, University Children's Hospital, Belgrade, Serbia; Pediatric Nephrology, Dialysis and Transplant Unit, Department of Woman and Child Health, Azienda Ospedaliera-University of Padova, Padova, Italy; Pediatric Nephrology Unit, Centre Hospitalier Universitaire de Bordeaux, Université de Bordeaux, Bordeaux, France; Division of Pediatric Nephrology, Center for Pediatrics and Adolescent Medicine, Heidelberg University Hospital, Heidelberg, Germany; Department of Pediatric Kidney Liver, Metabolic and Neurological Diseases, Hannover Medical School, Hannover, Germany; Department of Pediatrics, Division of Gastroenterology, Nephrology and Metabolic Medicine, Charité - Universitätsmedizin Berlin, corporate member of Freie Universität Berlin, and Humboldt-Universität zu Berlin, Berlin, Germany; Division of Pediatric Nephrology, Center for Pediatrics and Adolescent Medicine, Heidelberg University Hospital, Heidelberg, Germany; Department of Pediatric Kidney Liver, Metabolic and Neurological Diseases, Hannover Medical School, Hannover, Germany

**Keywords:** cardiovascular morbidity, chronic kidney disease, growth hormone, Klotho, pulse wave velocity

## Abstract

**Background:**

Chronic kidney disease (CKD) is characterized by low levels of the anti-aging protein α-Klotho and accelerated cardiovascular (CV) morbidity. Short-term treatment with growth hormone (GH) was shown to enhance soluble Klotho (sKlotho), the circulating form of α-Klotho, and endothelial function in patients with CKD. We hypothesized that long-term GH treatment in pediatric patients with CKD improves sKlotho levels and CV morbidity.

**Methods:**

We performed a case-cohort study within the Cardiovascular Comorbidity in Children with Chronic Kidney Disease (4C) study including 101 children with CKD stages 3–5 treated with and without GH. Patients were assessed for serum sKlotho, intact fibroblast growth factor 23 (iFGF23), somatomedin insulin-like growth factor 1 (IGF1), pulse wave velocity (PWV), carotid intima thickness (cIMT), and left ventricular mass index (LVMI) at two time points 12 months apart.

**Results:**

GH-treated patients showed higher sKlotho (Δ1.2 SD) and IGF1 (Δ1.5 SD) *z*-scores, and lower PWV *z*-scores (Δ −0.9 SD) compared to controls (each *P* < .01), both at baseline and after 12 months of follow up. iFGF23 and cIMT *z*-scores, LVMI, and progression of CKD did not differ between groups. In the multivariable analysis, sKlotho z-scores associated with GH treatment, IGF1 and iFGF23 *z*-scores (each *P* < .01). PWV *z*-scores associated with GH treatment, diastolic blood pressure, and parathyroid hormone levels, while cIMT *z*-score and LVMI associated with diastolic blood pressure and hemoglobin only (each *P* < .05).

**Conclusions:**

Long-term GH treatment is associated with reduced PWV in children with CKD, which is at least partly related to GH/IGF1-induced upregulation of sKlotho.

## Graphical Abstract

**Graphical Abstract:**
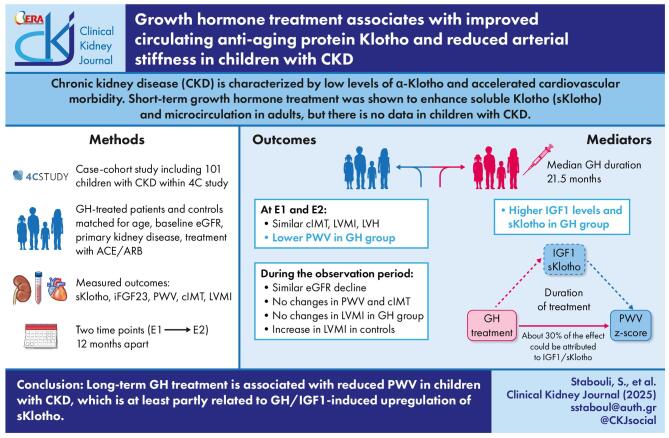

KEY LEARNING POINTS **What was known:**

- Chronic kidney disease (CKD) is characterized by low levels of the anti-aging protein α-Klotho and accelerated cardiovascular morbidity.
- Upregulation of Klotho positively affects the cardiovascular system by enhancing fibroblast growth factor 23 (FGF23) mediated nitric oxide release from small vessels and reducing oxidative stress *in vitro*.
- Short-term growth hormone (GH) treatment was shown to enhance soluble Klotho (sKlotho), the circulating form of α-Klotho, and endothelial function in adult patients with CKD stages 3–5.

**This study adds:**

- The beneficial effects of long-term GH treatment often used to treat CKD-associated growth failure in children with CKD, may extend beyond height, and encompass cardiovascular outcomes.
- In this case-cohort study, children with CKD stages 3–5 on long-term GH treatment showed improved levels of sKlotho compared to controls and the difference remained significant during the observation period. The expected decline of sKlotho observed in parallel to declining eGFR in controls was blunted in GH-treated patients.
- Pulse wave velocity was reduced in GH-treated patients compared to controls indicating improved vascular stiffness. Pulse wave velocity associated with GH treatment, IGF1 and sKlotho, suggesting that the beneficial effects of GH treatment on the vasculature are at least partly related to IGF1-induced upregulation of sKlotho levels.

**Potential impact:**

- GH treatment can affect intermediate cardiovascular outcomes beyond growth, i.e. through increased IGF1/Klotho synthesis leading to reduced arterial stiffness and therefore, has a potential role in the prevention of accelerated cardiovascular morbidity in pediatric CKD patients.
- Current policy should be strengthened to ensure that all short children with CKD stages 3–5 are offered GH treatment in line with current guidelines.

## INTRODUCTION

Chronic kidney disease (CKD) is characterized by low levels of the anti-aging protein α-Klotho and accelerated cardiovascular (CV) morbidity including left ventricular hypertrophy (LVH), vascular stiffness and atherosclerosis, which are observed even in childhood in up to 50% of patients with CKD stages 3–5 [1, 2]. α-Klotho is a 130-kDa membrane-bound protein mainly expressed in the kidney acting as cofactor for the phosphaturic hormone fibroblast growth factor 23 (FGF23). FGF23 binds to the FGF receptor 1-α-Klotho complex resulting in downregulation of the sodium-dependent phosphate transporters NaPi2a and NaPi2c and suppression of 1,25-dihydroxyvitamin D_3_ [1,25 (OH)_2_D_3_] synthesis in proximal renal tubules and consecutive enhanced phosphate excretion and decreased 1,25 (OH)_2_D_3_ levels [3, 4]. α-Klotho occurs in two forms, a membrane-bound aKlotho, and soluble Klotho (sKlotho), which arises from proteolytic cleavage of the extracellular domain of the transmembrane protein. Reduced levels of sKlotho are associated with premature aging, impaired kidney function, and CV disease in the general population and patients with CKD [5, 6, 7].

Growth hormone (GH) is thought to stimulate calcitriol production and the phosphaturic and vascular actions of FGF23 by upregulation of FGF23/Klotho axis [8]. This is based on the observation that short-term GH treatment and endogenous GH excess enhance serum sKlotho concentrations in healthy people, patients with CKD stage 3, and acromegalic patients [8, 9]. Experimental studies indicate that upregulation of Klotho positively affects the CV system by enhancing FGF23 mediated nitric oxide (NO) release from small vessels and reducing oxidative stress, leading to enhanced microvascular function [10]. Likewise, a 7-day treatment with GH in healthy people and adult patients with CKD stages 3–5 increased microcirculation under resting conditions, which was paralleled by reduced total vascular resistance [11].

Therefore, we hypothesized that long-term GH therapy given for treatment of CKD-associated short stature improves serum sKlotho concentrations, and CV morbidity in pediatric CKD patients. To this aim we performed a case-cohort study within the Cardiovascular Comorbidity in Children with Chronic Kidney Disease (4C) study including 101 children with CKD stages 3–5 treated with and without concomitant GH treatment. Patients were assessed for serum concentrations of sKlotho, intact FGF23 (iFGF23), 1,25 (OH)_2_D_3_, and the somatomedin insulin-like growth factor 1 (IGF1), and major surrogate markers for CV damage including pulse wave velocity (PWV), LV mass index (LVMI), and carotid intima media thickness (cIMT) at two time points 12 months apart.

## MATERIALS AND METHODS

### Participants and study design

We performed a case-cohort study within the 4C study [1]. The 4C study is a European prospective observational study in pediatric CKD patients with an estimated glomerular filtration rate (eGFR) between 10 and 60 ml/min per 1.73 m^2^ and an age at study inclusion between 6 and 17 years, investigating CV status and a comprehensive set of clinical and laboratory parameters in 6-month intervals. The indication for GH treatment was met by the attending physicians for treatment of CKD-associated growth failure [12]. In 668 patients enrolled in the 4C-study a complete set of yearly CV assessments were available in 104 children receiving GH treatment. From the latter group we identified 34 cases (GH group) with adequate plasma samples for whom 67 non-GH-treated controls (one control was matched for two cases) were selected (Supplementary file 1). Matching was performed in 1:2 ratio for age, baseline eGFR ($\pm $5 ml/min/1.73 m^2^), primary kidney disease, and treatment with inhibitors of the renin angiotensin system (ACE/ARB). Exclusion criteria were start of dialysis or kidney transplantation during the study period. Written informed consent was obtained from all caregivers and children as appropriate. The study was conducted according to the Declaration of Helsinki guidelines, and all local ethics committees approved the study.

### PWV

Aortic PWV was assessed using the oscillometric Vicorder device (SMT medical, Würzburg, Germany). Settings and measurement conditions were as previously reported [13, 14]. The distance from the suprasternal notch to the femoral recording point via the umbilicus was used as path length. The measured values were transformed to *z*-scores per height using reference values established by our group [13].

#### cIMT

Ultrasound examination was performed per the Mannheim carotid IMT consensus [15] using an 8-MHz annular array ultrasound imaging system (Siemens Acuson P50 Ultrasound system, Software version 2.1) with integrated digital image evaluation software (Syngo US Workplace, Siemens Medical Solutions, USA Inc.). Five segments of the left and right common carotid artery in the anterior–posterior projection were examined and averaged. All values were transformed to *z*-scores per height [16].

### LVMI

Two-dimensional echocardiography was performed to assess LV volume as previously described [17]. The LVMI was calculated by the Devereux Equation and indexed to the allometric height in meters raised to the power of 2.7 [18]. The sex- and age-specific LVMI partition values of Khoury *et al.* were used to define LVH [19].

### Clinical assessments and laboratory analyses

For each visit, the following variables were extracted from the 4C database: primary kidney disease, weight, height, body mass index (BMI), office systolic and diastolic blood pressure (BP), duration and dose of GH medication, treatment with active and native vitamin D (yes/no), antihypertensive medication (yes/no), and ACEi/ARBs (yes/no); and laboratory parameters including hemoglobin, intact parathyroid hormone (iPTH), calcium, phosphate, bicarbonate, albumin, total cholesterol, high density lipoprotein (HDL), low density lipoprotein (LDL), and triglycerides, which were measured centrally by standard laboratory techniques. eGFR was calculated using the revised Schwartz formula [20]. For each visit plasma and serum in the biobank was used for further laboratory measurements including sKlotho, iFGF23, 1,25 (OH)_2_D_3_, and IGF1. Enzyme-linked immunosorbent assay kits were used for serum sKlotho (#27998, Immuno-Biological Laboratories, Japan), plasma iFGF23 (#60–6600, Immutopics, San Clemente, CA, USA), plasma IGF1 (# MD58011, IBL International, Hamburg, Germany), and serum 1,25 (OH)_2_D_3_ (# AC-62F1, Immunodiagnostic System, East Boldon, UK) according to the manufacturer's protocols. All samples were measured in duplicate. Inter-/intra-assay coefficients of variation were <8%.

### Statistical analyses

The CV endpoints of the study were the comparison of PWV, cIMT, and LVMI at first observation (E1) and last observation (E2) and their changes from E1 to E2 in patients with and without concomitant GH treatment. Biochemical outcomes tested were sKlotho, iFGF23, IGF1 at E1 and E2, and their changes during the study period. Age and sex adjusted *z*-scores were calculated for height, BMI, office systolic and diastolic BP, IGF1, sKlotho, and iFGF23 [21-23].

Data are given as mean ± SD/SE or median (interquartile range, IQR) depending on the presence of normal distribution assessed by Shapiro–Wilk test. Comparisons between groups were performed using chi square, *t*-test and paired *t*-test, or non-parametric tests (Mann–Witney *U*, Wilcoxon, or McNemar tests) as appropriate. Spearman correlation and regression analysis were used to test for associations of CV and biochemical outcomes with clinical and laboratory variables. We used generalized estimating equations (GEE) models to account for matching in the design and to evaluate associations between outcomes and variables including age, sex, GH treatment (case versus control), native and active vitamin D supplementation (yes versus no), BMI *z*-score, systolic/diastolic BP *z*-score, total cholesterol, HDL, LDL, triglycerides, hemoglobin, serum albumin, albumin corrected calcium, phosphate, iPTH, bicarbonate, 1,25 (OH)_2_D_3_, IGF1 *z*-score, and eGFR. Multivariable models were built with candidate independent variables showing a *P* value of <.10 in the univariate analysis, to account for residual confounding. All models were adjusted for age and sex. In addition, bootstrap mediation analyses was used to examine the effect of IGF1 and sKlotho *z*-scores on PWV *z*-scores, and to examine possible mediation to the effect of GH treatment on PWV *z*-score [24]. The exploratory statistical analysis of all results, as well as correlations with clinical and laboratory parameters with CV endpoints were performed using SPSS 27 software. *P* values <.05 were considered statistically significant.

## RESULTS

### Patient characteristics and general laboratory parameters at first observation

**Figure 1:**
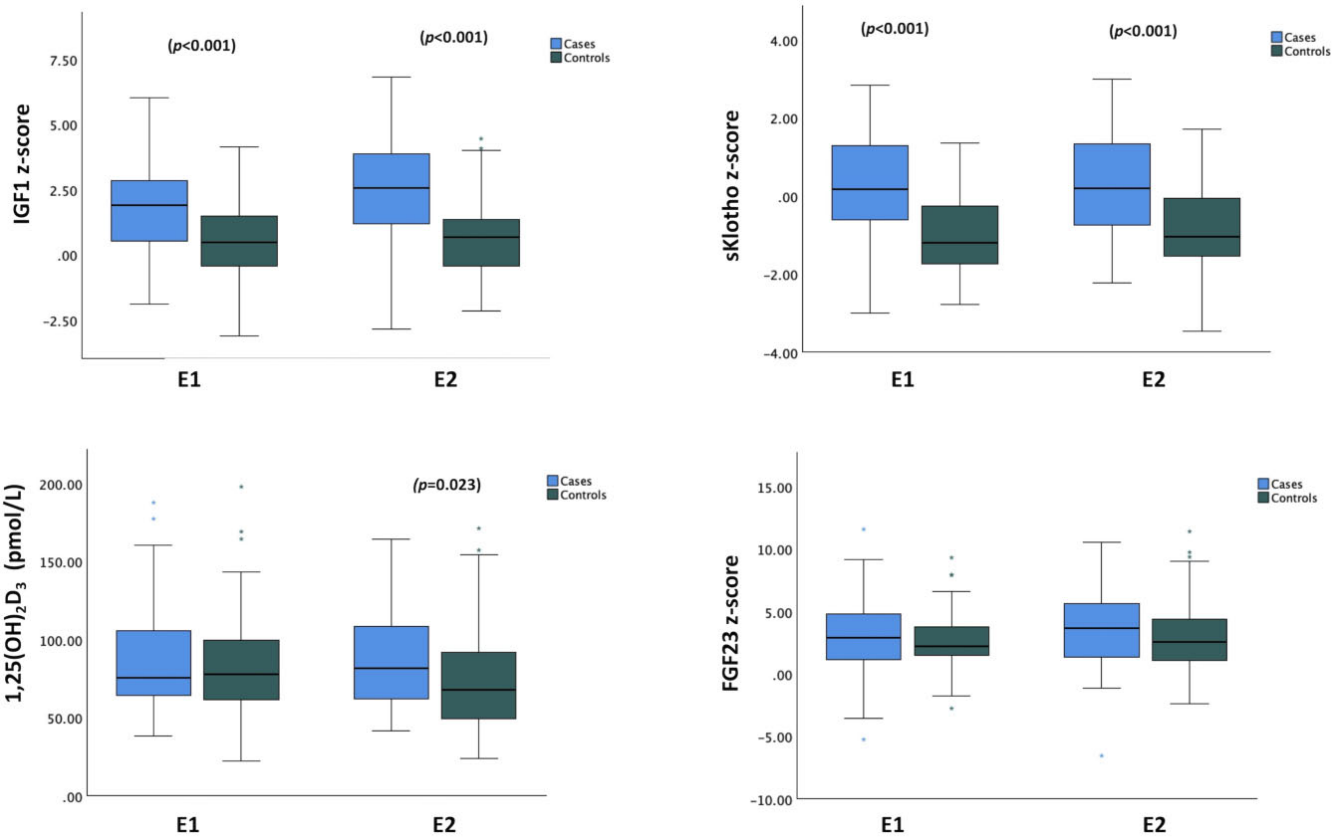
Serum IGF1, soluble Klotho, 1,25(OH)_2_D_3_, and iFGF23 concentrations at the first (E1) and second (E2) observations in GH-treated patients (cases) and controls.

Mean age of the study population was 12.1 ± 2.8 years, and median duration of GH treatment in the GH group amounted to 21.5 months (IQR, 7.0–56.7 months). At the time of first observation (E1), GH-treated and untreated patients did not significantly differ with respect to anthropometric parameters, median eGFR, BP values, hemoglobin, serum phosphate, iPTH, 1,25 (OH)_2_D_3_, and lipids values (Table 1). GH-treated patients showed significantly higher bicarbonate and albumin corrected-Ca levels compared to controls. As expected IGF1 *z*-scores were significantly higher in the GH group compared to controls (mean difference ± SE, E1: 1.31 ± 0.36, E2: 1.76 ± 0.40, each *P* < .001) (Fig. 1). sKlotho *z*-scores were significantly higher in GH-treated patients (mean difference ± SE, E1: 1.19 ± 0.28, E2: 1.08 ± 0.27, each *P* < .001), while iFGF23 *z*-scores did not differ between groups (Fig. 1). Native and active vitamin D were administered more frequently in GH-treated patients compared to controls, while there was no difference in the number of antihypertensive medications or the use of ACE/ARBs. Also, there was no significant change in the percentage of patients who were receiving antihypertensive medications in both groups between E1 and E2.

**Table 1:** Baseline characteristics, parameters of FGF23-Klotho-1,25(OH)_2_D_3_ axis, and cardiovascular measures in GH-treated pediatric patients with CKD stages 3–5 and matched controls.

Variable	GH group (*n* = 34)	Controls (*n* = 67)	*P*
Age, years	12 (3)	12 (3)	.2
Male sex, %	28 (82)	49 (73)	.3
CAKUT *N* (%)	28 (82)	56 (84)	.9
Height, *z*-score	−1.23 (1.07)	*−*1.44 (1.31)	.4
BMI, *z*-score	0.16 (1.01)	0.25 (1.06)	.7
SBP, *z*-score	0.59 (0.96)	0.72 (1.15)	.6
DBP, *z*-score	0.33 (0.95)	0.60 (0.86)	.2
eGFR, ml/min/1.73 m^2^	24 (10)	24 (10)	.9
Hemoglobin, g/dl	11.55 (1.31)	11.60 (1.54)	.9
Total cholesterol, mg/dl	178.5 (37.7)	183.8 (43.3)	.5
LDL, mg/dl	100.9 (35.2)	105.0 (35.9)	.6
HDL, mg/dl	49.8 (11.8)	50.0 (12.6)	.9
Triglycerides, mg/dl	156.8 (63.3)	138.8 (84.4)	.3
Bicarbonate, mmol/l	22.8 (2.5)	21.4 (3.1)	.03
Albumin, g/l	41.2 (4.5)	40.1 (3.8)	.2
Calcium, mmol/l	2.38 (2.24 to 2.55)	2.31 (0.19 to 2.43)	.04
Phosphate, mmol/l	1.59 (1.41 to 1.69)	1.57 (1.35 to 1.74)	.7
iPTH, pmol/l	11.66 (7.34 to 17.85)	12.40 (8.25 to 29.20)	.2
IGF1, *z*-score	1.90 (0.48–2.87)	0.48 (*−*0.54 to 1.49)	<.001
sKlotho, *z*-score	0.16 (*−*0.77 to 1.33)	*−*1.18 (*−*1.74 to *−*0.22)	<.001
iFGF23, *z*-score	2.94 (1.15 to 5.09)	2.25 (1.48 to 5.18)	.4
1,25(OH)_2_D_3_, pmol/l	76 (64 to 107)	78 (61 to 100)	.5
Native vitamin D, *N* (%)	15 (44.1)	11 (16.4)	.003
Active vitamin D, *N* (%)	27 (79.4)	39 (58.2)	.03
Antihypertensive medication, *N* (%)	18 (52.9)	41 (61.2)	.4
GH duration, months	21.5 (7.0–56.75)	n.a.	n.a
GH cumulative dose, mg	703 (250–2142)	n.a.	n.a.
PWV, *z*-score	*−*0.54 (1.52)	0.35 (1.59)	.01
LVMI, g/m^2.7^	45.93 (10.26)	45.85 (13.04)	.9
cIMT, *z*-score	1.64 (1.29)	1.73 (1.42)	.8

Data are given as mean (SD) or median (interquartile range, IQR) or *N* (%) as appropriate; n.a., not applicable.

Abbreviations: SBP, systolic BP, DBP, diastolic BP.

### Kidney function, FGF23-Klotho-1,25 (OH)_2_D_3_ axis and associated factors during study period

The median change in eGFR during the observation period was −2.21 ml/min/1.73 m^2^ (IQR, −5.07 to 0.32, *P* < .001) in the whole patient cohort, and did not differ between groups (GH group −1.08 ml/min/1.73 m^2^ (IQR, −4.5 to 0.69); controls −2.26 ml/min/1.73 m^2^ (IQR, −5.09 to −0.09), *P* = .5). During the observation period, serum IGF1 levels and height *z*-score significantly increased in GH-treated patients, but not in controls; sKlotho *z*-score, iFGF23 *z*-score, and 1,25 (OH)_2_D_3_ concentrations did not significantly change within patient groups, while iPTH levels increased in controls only (Table 2). Serum sKlotho *z*-score and 1,25 (OH)_2_D_3_ levels at E2 were significantly higher in GH-treated patients compared to controls (Fig. 1 and Table 2).

**Table 2:** Changes in clinical and biochemical parameters, and cardiovascular measurements during the study period in GH-treated patients and matched controls.

	GH group (*n* = 34)			Controls (*n* = 67)		
Variable	Start (E1)	End (E2)	*P* value	Start (E1)	End (E2)	*P* value
Height, *z*-score	−1.23 (1.07)	−1.02 (1.04)	<.001	−1.44 (1.31)	−1.50 (1.37)	.7
BMI, *z*-score	0.16 (1.01)	0.03 (1.07)	.089	0.25 (1.06)	0.29 (1.06)	.3
SBP, *z*-score	0.59 (0.96)	0.50 (1.07)	.622	0.72 (1.15)	0.74 (1.11)	.9
DBP, *z*-score	0.33 (0.95)	0.30 (0.98)	.894	0.60 (0.86)	0.61 (0.99)	.9
eGFR, ml/min/1.73 m^2^	19.8 (14.8–32.9)	16.1 (13.7–31.4)	.039	22.2 (16.4–31.9)	19.7 (13.6–28.5)	<.001
Hemoglobin, g/dl	11.55 (1.34)	11.77 (1.68)	.66	11.58 (1.5)	11.50 (1.07)	.7
iPTH, pmol/l	11.6 (7.3–18.8)	14.8 (6.2–21.3)	.102	12.4 (8.2–29.2)	17.5 (9.3–32.4)	.006
Calcium, mmol/l	2.38 (2.24–2.55)	2.43 (2.31–2.55)	.574	2.31 (2.19–2.43)	2.37 (2.26–2.53)	.001
Phosphate, mmol/l	1.59 (1.41–1.69)	1.65 (1.21–1.84)	.521	1.57 (1.16–1.74)	1.61 (1.35–1.73)	.3
IGF1, ng/ml	456.6 (232.3)	586.5 (288.2)	.019	335.3 (174.0)	371.0 (197.8)	.08
IGF1, *z*-score	1.90 (0.48–2.87)	2.57 (1.17–3.93)	.079	0.48 [(−0.54) −1.49]	0.67 (−0.48 to 1.38)	.3
sKlotho, pg/ml	1822 (1499–3322)	1599 (983–3142)	.845	977 (752–1652)	946 (699–1419)	.9
sKlotho *z*-score	0.16 (−0.77 to 1.33)	0.21 [(−0.77 to 1.38)	.754	−1.18 (−1.74 to −0.22)	−1.04 (−1.54 to 0.08)	.4
iFGF23, pg/ml	124 (67–223)	161 (72–315)	.640	97 (74–168)	110 (66–218)	.2
iFGF23 *z*-score	2.94 (1.15–5.09)	3.68 (1.37–7.08)	0.588	2.25 (1.48–5.18)	2.60 (1.10–4.49)	.1
1,25(OH)_2_D_3_, pmol/l	76 (64–107)	82 (62–1099)	.952	78 (61–100)	68 (49–93)	.08
PWV, *z*-score	−0.54 (1.52)	−0.52 (1.17)	.862	0.35 (1.59)	0.35 (1.40)	.7
LVMI, g/m^2.7^	44.8 (38.1–53.5)	46.4 (38.9–56.2)	.980	44.2 (37.7–50.0)	45.1 (39.6–56.2)	.05
LVH (%)	67.6	76.5	.02	67.2	79.1	<.001
cIMT, *z*-score	1.64 (1.29)	1.95 (1.08)	.137	1.73 (1.42)	1.85 (0.77)	.4

Data are presented as mean (SD) or median (IQR). Abbreviations: CAKUT: congenital anomalies of the kidneys and urinary tract, SBP, systolic BP, DBP, diastolic BP.

**Figure 2:**
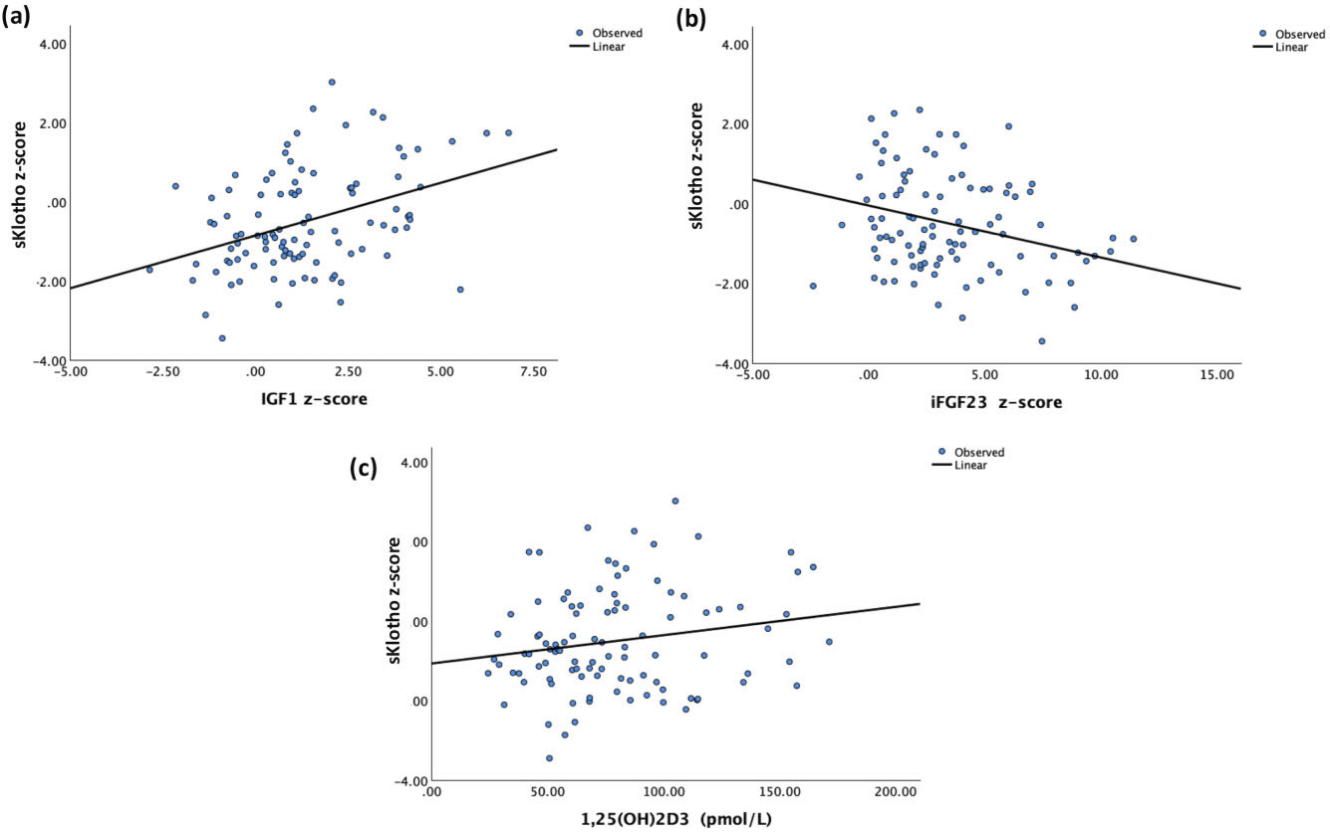
Soluble Klotho *z*-scores as a function of (a) IGF1 *z*-scores, (b) iFGF23 *z*-scores, and (c) 1,25(OH)_2_D_3_ levels at the first observation (E1).

**Figure 3:**
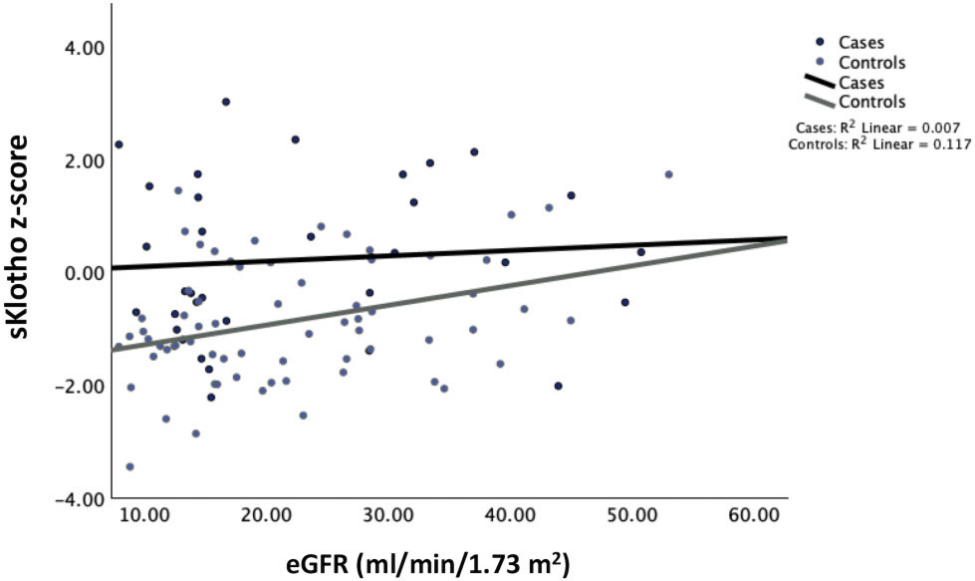
Changes in sKlotho *z*-score as a function of eGFR (ml/min/1.73 m^2^) during the study period in GH-treated patients (cases) and controls.

In the total study population, sKlotho *z*-score was positively associated with eGFR (E2 *R*^2^ = 0.051, *P* < .05), IGF1 *z*-score (E1 *R*^2^ = 0.061, *P* < .05; E2 *R*^2^ = 0.153 *P* < .001), 1,25 (OH)_2_D_3_ (E1 *R*^2^ = 0.087, *P* = .003; E2 *R*^2^ = 0.038, *P* = .05) levels, and negatively associated with iFGF23 z-score (E2 *R*^2^ = 0.093, *P* = .002) (Fig. 2a-c), and total cholesterol levels (E1 *R*^2^ = 0.054, *P* < .05; E2 *R*^2^ = 0.058, *P* < .05). The slope of the regression line between sKlotho and eGFR at the final observation (E2) was significant only in controls (*β* = 1.035, 95%CI 1.01–1.06, *P* = .005, for interaction between control group and final eGFR) (Fig. 3).

In the GEE multivariable model, sKlotho *z*-sores during the observation period were significantly associated with GH treatment, IGF1 *z*-score, iFGF23 *z*-score, and total cholesterol levels, while iFGF23 *z*-scores were associated with eGFR, male sex, calcium, and sKlotho *z*-score (Table 3).

**Table 3:** Associations of sKlotho, iFGF23 in the total study population during the observation period analyzed by multivariate GEE models.

Dependent variable	Parameter	β	95% CI	*P*
sKlotho *z*-score	GH treatment (yes vs no)	2.238	1.392–3.597	<.001
	IGF1 *z*-score	1.194	1.072–1.330	.001
	iFGF23 *z*-score	0.898	0.841–0.960	.002
	1,25(OH)_2_D_3_, pmol/l	1.005	1.000–1.009	.06
	Total cholesterol, mg/dl	0.996	0.991–1.000	.05
	eGFR, ml/min/1.73 m^2^	1.007	0.985–1.030	.5
iFGF23 *z*-score	Sex (male vs female)	3.885	1.620–9.318	.002
	eGFR, ml/min/1.73 m^2^	0.955	0.918–0.993	.02
	Calcium, mmol/l	51.321	8.093–325.442	<.001
	sKlotho *z*-score	0.686	0.472–0.998	.05

Covariates were included in the multivariate analysis if *P* < .1 in univariate models.

### PWV, LVMI, and cIMT and associated factors during study period

**Figure 4:**
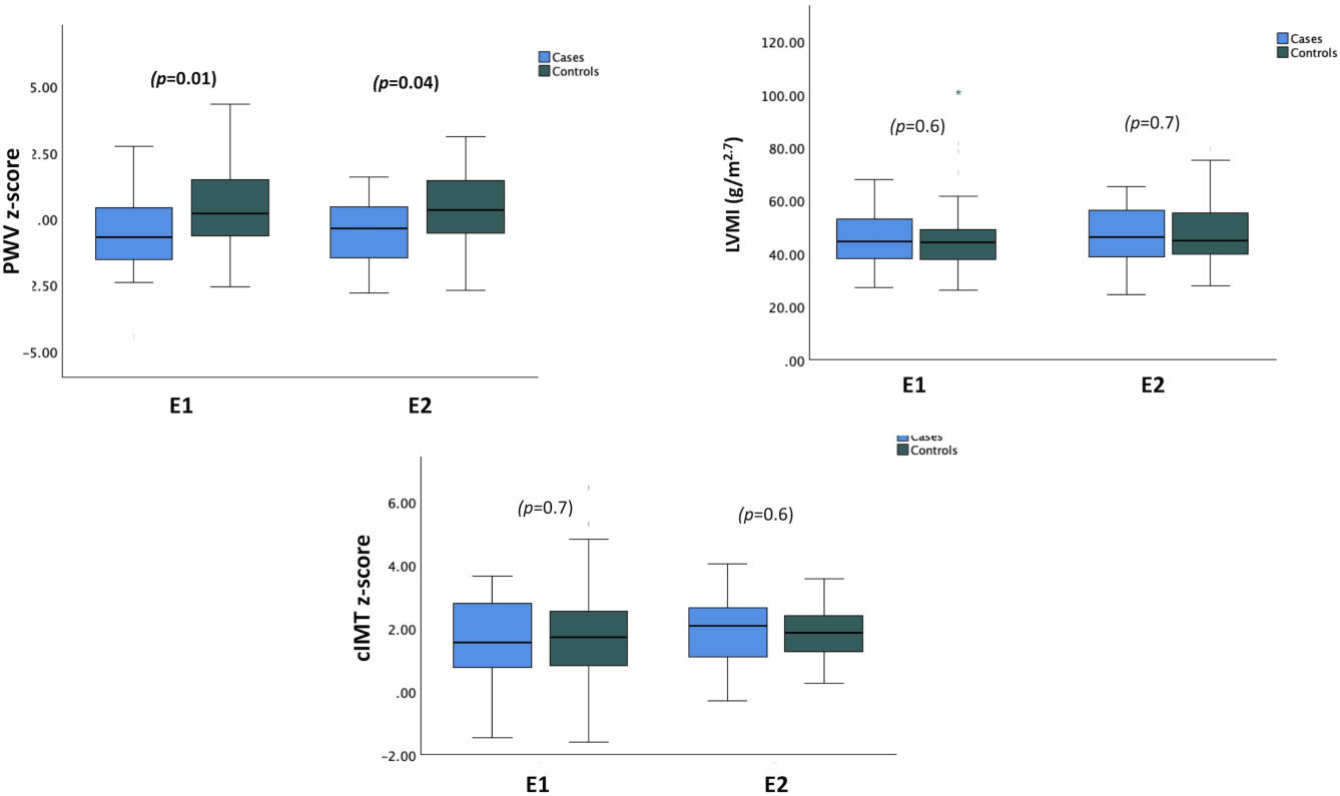
Cardiovascular endpoints at baseline and at the end of the observation period in GH-treated patients and controls.

PWV *z*-scores were significantly lower in GH-treated patients compared to controls at both the first and last observations (mean difference ± SE, E1 −0.89 ± 0.33; E2 −0.88 ± 0.29, both *P* < .005). PWV *z*-scores did not change significantly during the observation period, irrespective of GH treatment (Table 2, Fig. 4). In contrast, LVMI values, LVH prevalence, and cIMT *z*-scores did not differ between groups (Table 1, Fig. 4). There was also no difference in the prevalence of LVH between GH-treated patients and controls at E2 (Supplementary file 3). However, during the observation period LVMI significantly increased in controls only (Table 2). Numerically, but not statistically significant, more patients in the control group progressed to LVH, which is in line with the significantly increase in LVMI observed in the control group. Finally, no significant changes were noted for cIMT *z*-scores, irrespective of GH treatment.

**Figure 5:**
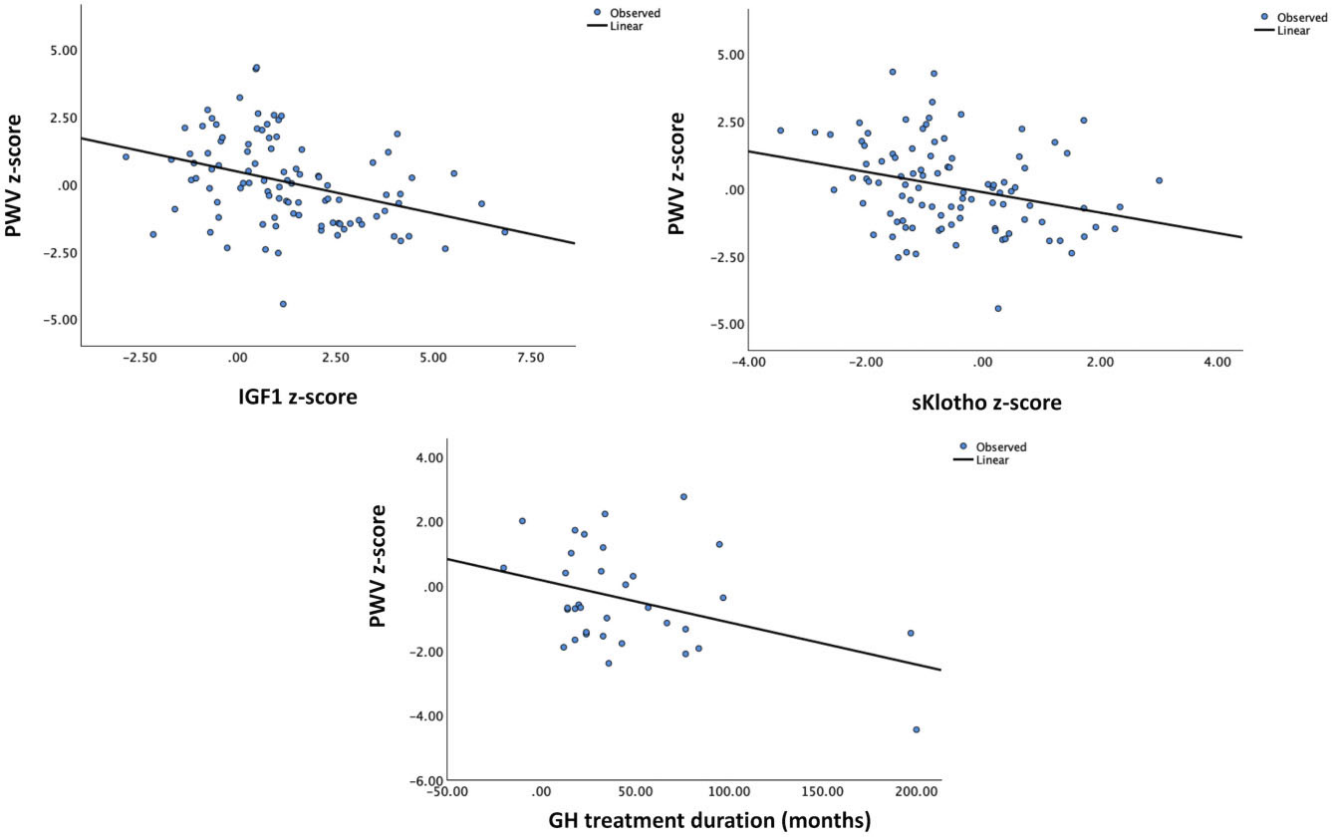
PWV *z*-scores in GH-treated patients as a function of IGF1 and sKlotho *z*-scores, and duration of GH treatment at the first observation (E1).

In the total study population, PWV *z*-score was inversely associated with IGF1 *z*-score (E1 *R*^2^ = 0.130, *P* < .001; E2 *R*^2^ = 0.095, *P* = .003), and sKlotho *z*-score (E1 *R*^2^ = 0.086, *P* = .004) (Fig. 5), and positively associated with systolic BP *z*-score (E1 *R*^2^ = 0.134, *P* < .001; E2 *R*^2^ = 0.157, *P* < .001), diastolic BP *z*-scores (E1 *R*^2^ = 0.206, *P* < .001; E2 *R*^2^ = 0.304, *P* < .001) and iPTH levels (E2 *R*^2^ = 0.084, *P* < .01). In GH-treated patients, PWV *z*-score was inversely associated with duration of GH treatment at baseline (E1 *R*^2^ = 0.160, *P* < .05). In GEE multivariable model, PWV *z*-score during the observation period were significantly associated with GH treatment, diastolic BP *z*-score, and iPTH levels (Table 4). LVMI associated only with hemoglobin levels in GEE model (Table 4). cIMT *z*-score was associated with BP values in GEE models (Table 4).

**Table 4:** Associations of PWV *z*-score, cIMT *z*-score, and LVMI in the total study population during the observation period analyzed by multivariate GEE models.

Outcome	Parameter	β	95% CI		*P*
PWV *z*-score	GH treatment (yes vs no)	0.579	0.358	0.938	.03
	iPTH, pmol/l	1.008	1.001	1.014	.02
	SBP *z*-score	0.987	0.758	1.286	.9
	DBP *z*-score	2.098	1.531	2.875	<.001
LVMI	GH treatment (yes vs no)	0.647	0.013	31.250	.8
	eGFR, ml/min/1.73 m^2^	0.871	0.754	1.005	.6
	Hemoglobin, g/dl	0.335	0.130	0.862	.02
	BMI *z*-score	3.199	0.827	12.371	.09
cIMT *z*-score	GH treatment (yes vs no)	1.087	0.785	1.504	.6
	SBP *z*-score	1.043	0.868	1.254	.6
	DBP *z*-score	1.290	1.038	1.603	.02

Covariates were included in the multivariate analysis if *P* < .1 in univariate models. Multivariate models are adjusted for age and sex.

Abbreviations: SBP, systolic BP, DBP, diastolic BP.

Mediation analysis showed that the effect of GH treatment on PWV *z*-score is mediated by sKlotho and IGF1 *z*-score with significant indirect effects coefficients 0.28, SE 0.14, 95% confidence interval (CI) 0.04–0.59 and 0.29, SE 0.17, 95%CI 0.02–0.71, respectively (Supplementary file 2).

## DISCUSSION

In this case-cohort study, we investigated our hypothesis that long-term GH treatment in children with CKD stages 3–5 is associated with improved serum sKlotho and CV morbidity with respect to reduced arterial stiffness. The expected decline of sKlotho observed in parallel to declining eGFR in CKD controls [25] was blunted in GH-treated patients. sKlotho levels were already increased at baseline in patients on long-term GH treatment and the difference remained significant during the observation period. Patients treated with GH also presented with lower PWV *z*-scores at all time points compared to controls indicating reduced vascular stiffness. PWV *z-*scores associated with the duration of GH treatment, IGF1, and sKlotho *z*-scores suggesting that the beneficial effects of GH treatment on the vasculature are at least partly related to IGF1-induced upregulation of sKlotho levels.

The GH/IGF1 axis may exert several effects on the kidney including kidney glomerular hemodynamics, tubular sodium and water, phosphate, and calcium handling, as well as renal synthesis of 1,25 (OH)_2_D_3_ and the anti-aging protein α-Klotho [26]. In the present study GH treatment had no adverse effects on CKD progression supporting previous observations in GH-treated CKD patients [27, 28]. Chronic kidney disease-associated GH-insensitivity and/or generally limited renal reserve capacity [26], may explain the comparable decline in eGFR in both patient groups in the study. On the other hand, recent studies indicating that the deficiency of α-Klotho could contribute to GH resistance observed in CKD, the lack of α-Klotho might be preventing proper GH signaling in the kidneys [26, 29].

Previous studies showed that GH treatment increases sKlotho levels both in GH-deficient patients with normal kidney function but also in adults and children with CKD [9, 30-32]. In the present study, higher IGF1 levels associated with significantly higher sKlotho levels, which is in line with observations in healthy children, and GH-sufficient and GH-deficient patients [26, 33], and further supports the concept that the stimulating actions of GH on sKlotho may at least partly mediated by the somatomedin IGF1. Moreover, patients treated with GH had higher levels of sKlotho during the whole study period despite similar levels of iFGF23, while sKlotho decreased with CKD progression in GH-untreated patients only. Thus, GH treatment may attenuate the adverse effect of decreasing eGFR on renal α-Klotho synthesis and thus, sKlotho levels, likely mediated by IGF1, and independently of FGF23 [29].

There is a growing body of evidence that FGF23/Klotho axis has a regulating role in arterial remodeling in patients with CKD [34]. Intimal-media calcifications are a major feature of CKD-associated mineral and bone disorder (CKD-MBD) resulting in vascular stiffness which can be detected by increased PWV. High FGF23 exerts its pathological function on vessels in a Klotho-dependent manner with the disturbed balance between FGF23 and its cofactor α-Klotho contributing to the increased calcification in CKD [35]. Decreased sKlotho levels have been independently associated with arterial stiffness in adult CKD patients [36]. Beyond vascular calcification, sKlotho may exert protective effects on the endothelium and reduce endothelial dysfunction by regulating NO availability [37]. Thus, by increasing NO production resulting in arterial vasodilation, sKlotho reverts the FGF23-induced vasoconstriction observed in the state of Klotho deficiency [10].

These mechanistic insights may explain the lower PWV in GH-treated patients, while the lack of a significant association between cIMT and GH treatment suggest that different mechanisms and not mediated GH effects are responsible for increases in cIMT. In addition, 1,25 (OH)2D3 levels were significantly higher in the GH-treated patients compared to the controls, which is most likely due to the stimulating effects of GH on renal calcitriol synthesis [38]. Since high 1,25 (OH)2D3 levels are associated with increased cIMT in children with CKD, they could at least partially offset the positive effects of GH on the CV system in the present study [39]. Finally, the CV morbidity in CKD is a continuum under the detrimental effect of accumulating traditional and non-traditional risk factors. Each factor may predominantly affect different target organs, with GH treatment predominately affecting PWV-related CV morbidity.

The observed negative association between sKlotho and total cholesterol levels that remained significant in GEE models provides a further insight for the role of sKlotho in CV health. The association between serum sKlotho concentration and hyperlipidemia has been previously described in the adult general population using cross-sectional data from the NHANES 2007–2016 [40]. The potential mechanism by which Klotho negatively correlates with hyperlipidemia might involve anti-inflammatory effects, insulin resistance, and antioxidants. Several investigators discussed that therapeutic approaches to maintain or elevate the sKlotho level could improve arterial stiffness in CKD patients are warranted to be explored. In this concept GH treatment seems appealing, as it may improve CV outcome by attenuating adverse vascular alterations beyond its growth promoting effects in children with CKD.

In two pilot studies in adult CKD patients, short-term GH treatment improved CV risk factors and microcirculation [11, 41], while data on the potential effect of GH on uremic cardiomyopathy are lacking. Finally, the only RCT (OPPORTUNITY study) designed to assess all-cause and CV mortality in adult hemodialysis patients on GH treatment was terminated early due to slow recruitment and was underpowered for conclusions on hard outcomes [42]. Unfortunately, these studies in patients on GH treatment did not measure changes in the FGF23/Klotho system.

The high prevalence of LVH in childhood CKD has been previously highlighted in the 4C cohort [1]. A previous study in children with pre-dialysis CKD and after kidney transplantation did not find a link between FGF23 or sKlotho and LVMI, but the synergistic effect of both low sKlotho levels and high FGF23 levels have been associated with worse LV diastolic function [43]. sKlotho cardioprotective actions independent of FGF23 have been previously proposed in *in vitro* studies [44], but the results of the present study did not show significant associations between sKlotho and LVMI. GH treatment could also attenuate the adverse increases of LVMI with eGFR decline possibly by enhancing anthropometric measures such as height and BMI *z-*score, but also by maintaining lower cardiac afterload as indicated by lower PWV *z*-scores at all time points in GH-treated patients in the present study. The lack of association of LVMI with GH treatment may reflect the matching criteria for the study population that included matching for ACE/ARBs between GH-treated patients and controls. ACE/ARBs are known to have a direct effect on the heart independent of BP control, which could explain the lack of difference in LVMI between the GH-treated and non-treated patients. Still, LVMI levels remained stable in GH-treated patients at the end of the 12-month observation period. On the other hand, LVMI significantly increased in controls during the observation period.

The present study has several strengths and limitations. Limitations include the small population sample based on cohort data. The 4C study is an international multicenter study, and thus, it is possible that in an observational study, with ample geographical distribution, some differences in practices between centers could have affected the study results. Based on the study inclusion criteria only 34 GH-treated patients had all criteria, including available biosamples in the biobanking, included in the study. Moreover, biosamples before GH treatment initiation were not available to compare changes in sKlotho before and after GH treatment. Information about puberty including the Tanner stage was not available for the study participants. On the other hand, the present study was specifically designed to evaluate over a 12-month period the effects of GH on arterial stiffness and their sustainability in association to changes of FGF23/Klotho axis, and provided important insights on the interactions of GH/IGF1/Klotho system and CV health in CKD. Of note, the interpretation of the mediation analysis model suggests that IGF1 and sKlotho mediate the relationship between GH and PWV in a limited way within an observational context. An RCT comparing treated and untreated children with GH would not be possible to realize. We used the concept of “pragmatic clinical trials” and designed a controlled intervention in an observational study of a “real-world” cohort approach to promote research within real-world settings to yield clinically relevant results [45].

In conclusion, the present study shows a central role of GH treatment on mitigating arterial stiffness assessed by PWV and likely mediated by its stimulating actions on IGF1/sKlotho. The association of GH treatment with improved arterial stiffness remained significant in models including CKD-MBD parameters and CV risk factors, BP and BMI, may suggest its independent beneficial effect on CV morbidity in CKD patients. The data delivered by the present study may provide the basis of evidence for a future prospective multicenter study and stimulate initiatives for collaboration with networks and industry for further research.

## Supplementary Material

sfaf231_Supplemental_Files

## Data Availability

All datasets generated for this study provided by 4C under license/by permission. Individual participant data that underlie results reported in this article, after deidentification (text, tables, figures, and appendixes) will be shared on request to the corresponding author with permission of 4C.

## Appendix

### Collaborators

The following principal investigators contributed to the **4C Study Consortium**:

Gerard Cortina, Klaus Arbeiter, Jiri Dusek, Jerome Harambat, Bruno Ranchin, Michel Fischbach, Ariane Zaloszyc, Uwe Querfeld, Jutta Gellermann, Sandra Habbig, Max Liebau, Matthias Galiano, Rainer Büscher, Charlotte Gimpel, Matthias Kemper, Jun Oh, Anette Melk, Daniela Thurn-Valassina, Anke Doyon, Elke Wühl, Franz Schaefer, Ulrike John, Simone Wygoda, Nicola Jeck, Birgitta Kranz, Marianne Wigger, Francesca Mencarelli, Francesca Lugani, Sara Testa, Giovanni Montini, William Morello, Enrico Vidal, Elisa Benetti, Luisa Murer, Ciara Matteucci, Stefano Picca, Augustina Jankauskiene, Karolis Azukaitis, Aleksandra Zurowska, Ilona Zagozozon, Dorota Drodz, Tomasz Urasinski, Mieczyslaw Litwin, Anna Niemirska, Lukasz Obrycki, Maria Szczepanska, Ana Texeira, Amira Peco-Antic, Dusan Paripovic, Giacomo Simonetti, Guido Laube, Ali Anarat, Aysun K. Bayazit, Fatos Yalcinkaya, Esra Baskin, Nilgun Cakar, Oguz Soylemezoglu, Ali Duzova, Yelda Bilginer, Hakan Erdogan, Osman Donmez, Ayse Balat, Aysel Kiyak, Salim Caliskan, Nur Canpolat, Mahmut Civilibal, Cengiz Candan, Sevinc Emre, Alev Yilmaz, Harika Alpay, Gul Ozcelik, Sevgi Mir, Betul Sözeri, Ipek K. Bulut, Nejat Aksu, Onder Yavascan, Yilmaz Tabel, Pelin Ertan, Ebru Yilmaz, and Rukshana Shroff.
