# Supplementary material for: Growth hormone treatment associates with improved circulating anti-aging protein Klotho and reduced arterial stiffness in children with CKD
Source: Clin Kidney J. 2025 Jul 23;18(9):sfaf231. doi: 10.1093/ckj/sfaf231 (PMC12548030; doi:10.1093/ckj/sfaf231)
Supplement: sfaf231_Supplemental_Files [file sfaf231_Supplemental_Files.zip › R1_clean_Supplementary file 2.docx]

**Supplemental file 2.**

**Bootstrap mediation analyses**

Bootstrap mediation analyses using bootstrap conditional effects procedures (PROCESS version 3.5 for SPSS, by Andrew F. Hayes) was used to examine the effect of sKlotho z-score on PWV z-scores, and whether sKlotho z-score mediated the effect of GH treatment on PWV z-score at E1 (first observation). There was a significant indirect effect to the GH treatment on PWV z-score by sKlotho z-score (coefficient =0.28, SE 0.14, 95% confidence interval (CI) 0.04-0.59) suggesting at least partial mediation of GH treatment by sKlotho z-score on PWV z-score. About 30% of the effect of GH treatment on PWV z-score could be attributed to sKlotho z-score levels. The statistical model for the mediation analyses is illustrated in the figure below.

GH treatment (no/yes)

PWV z-score

c=0.89

p=0.009

b=-0.26

p=0.03

a=-0.96

p=0.003

sklotho z-score

GH treatment (no/yes)

c’=0.61

p=0.09

PWV z-score

Similarly, for IGF1 effects on PWV z-score at E1. About 16% of the effects of IGF1 z-score on PWV z-score could be attributed to sKlotho z-score partial mediation effects (coefficient =-0.05, SE=0.03, 95%CI=-0.13 to -0.002).

IGF1 z-score

PWV z-score

c =0.30

p=0.001

b=-0.24

p=0.06

a=0.24

p=0.001

sklotho z-score

c’=0.25

p=0.004

IGF 1 z score

PWV z-score

Then, we examined the effect of IGF1 z-score on PWV z-scores at E1, and whether IGF1 z- score mediated the effect of GH treatment on PWV z-score at E1.

GH treatment (no/yes)

PWV z-score

PWV z-score

IGF1 z-score

b= -0.15

p=0.05

c=0.89

p=0.009

a= -1.86

p<0.001

GH treatment (no/yes)

c’=0.58

p=0.07

There was a significant indirect effect to the GH treatment on PWV z-score by IGF1 z-score (coefficient =0.29, SE 0.17, 95%CI 0.02~~-~~0.71) suggesting that the effect of GH treatment on PWV z-score is at least partly mediated by IGF1 z-score.

**Reference**

Andrew F. (2013). Introduction to Mediation, Moderation, and Conditional Process Analysis: A Regression-Based Approach. New York, NY: The Guilford Press. Journal of Educational Measurement 2014; 51 (3):335-337.
