# Supplementary material for: Growth hormone treatment associates with improved circulating anti-aging protein Klotho and reduced arterial stiffness in children with CKD
Source: Clin Kidney J. 2025 Jul 23;18(9):sfaf231. doi: 10.1093/ckj/sfaf231 (PMC12548030; doi:10.1093/ckj/sfaf231)
Supplement: sfaf231_Supplemental_Files [file sfaf231_Supplemental_Files.zip › R1_Supplementary file 3.docx]

**Supplementary file 3.**

**Supplementary Table 3a.** Prevalence of Left Ventricular Hypertrophy (LVH) at E1 and E2. In both groups the prevalence of LVH significantly increased during the observation period. However, there was no difference in the prevalence of LVH between GH-treated and non-treated patients both at E1 and E2.

|  | **LVH** | |  |
| --- | --- | --- | --- |
|  | **GH group** | **Controls** | ***P (GH group vs controls)*** |
| **E1** | 23 (67.6%) | 45 (67.2%) | 0.9 |
| **E2** | 26 (76.5 %) | 53 (79.1%) | 0.8 |
| ***P* *(E1 vs E2)*** | 0.02 | <0.001 |  |

**Supplementary Table 3b.** Changes in LVH during the observation period (E1 to E2). A lower number of patients in the GH group progressed to LVH, while 3 patients presented regression to normal LVMI levels. Numerically, but not statistically significant, more patients in the control group progressed to LVH, which is in line with the significantly increase in LVMI observed in the control group as seen in table 2 of the main manuscript.

|  | **Regress to normal** | **Stable** | **Progress to LVH** |
| --- | --- | --- | --- |
| **GH group** | 3 (8.8%) | 25 (73.5%) | 6 (5.9%) |
| **Controls** | 3 (4.5 %) | 53 (79.1%) | 11 (10.5%) |
