# Supplementary material for: Growth hormone treatment associates with improved circulating anti-aging protein Klotho and reduced arterial stiffness in children with CKD
Source: Clin Kidney J. 2025 Jul 23;18(9):sfaf231. doi: 10.1093/ckj/sfaf231 (PMC12548030; doi:10.1093/ckj/sfaf231)
Supplement: sfaf231_Supplemental_Files [file sfaf231_Supplemental_Files.zip › Supplementary file 1. docx.docx]

**Supplementary file 1.**


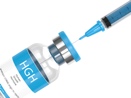
**Flow chart for the study population**

***Matching*** *in 1:2 ratio for:*

- age
- baseline eGFR ($\pm$5 ml/min/1.73m^2^)
- primary kidney disease
- treatment with inhibitors of the renin angiotensin system (ACE/ARB)

***Exclusion criteria:***

- start of dialysis or transplantation during the study period

***Inclusion criteria:***

- CKD stage 3-5
- two cardiovascular assessments (LVMI, PWV, cIMT) (E1, E2)
- time between E1 and E2 12 months (360 days $\pm$30 days)
- for each visit plasma available in the biobank

34 cases (GH group)

and 67 matched non-GH treated controls

104 children under GH treatment

668 patients enrolled in the 4C-study a complete set of yearly of cardiovascular assessments
